# Oral cancer awareness in North-Western Spain: a population-based study

**DOI:** 10.4317/medoral.24401

**Published:** 2021-06-20

**Authors:** Pablo Varela-Centelles, Juan Seoane, Yaima Ulloa-Morales, Ana Estany-Gestal, Andrés Blanco-Hortas, María J - García-Pola, Juan M - Seoane-Romero

**Affiliations:** 1 Praza do Ferrol Health Centre. EOXI Lugo, Cervo e Monforte. Galician Health, Lugo, Spain; 2 Department of Surgery and Medical-Surgical Specialities. School of Medicine and Dentistry. University of Santiago de Compostela, A Coruña, Spain; 3 Unit of Methodology of the Research. Health Research Institute of Santiago de Compostela, Spain; 4 Lugo University Hospital, Lugo, Spain; 5 Department of Surgery and Medical-Surgical Specialities. School of Medicine and Health Sciences, University of Oviedo, Spain

## Abstract

**Background:**

An early diagnosis depends greatly on patient awareness. Thus, the aim of this study was to investigate general awareness of oral cancer and knowledge about its risk factors, signs and symptoms.

**Material and Methods:**

Cross-sectional population-based survey of randomly selected respondents conducted from March 1, 2015 to 30 June 2016.

**Results:**

A total of 5,727 people entered the survey (response rate: 53%). When asked what cancers participants had heard about, 20.3% mentioned oral cancer. Regarding risk factors, tobacco was mentioned by 55.3% of the sample (n=3,169), followed by alcohol (12.5%; n=708), poor oral hygiene (10.8%; n=618), diet (6.5%; n=377), and genetics (4.5%; n=248).

**Conclusions:**

General population has low awareness of oral cancer with poor knowledge of risk factors and main alarm signs. In addition, individuals in the risk group scored lower values in the main variables analysed; even those highly educated showed insufficient awareness and knowledge of oral cancer. In these circumstances, there is clear need for educational interventions tailored to the target audience and aimed at increasing knowledge and awareness of oral cancer to promote primary prevention of oral cancer and minimising the time interval of patients with symptomatic oral cancer in their path to treatment.

** Key words:**Oral cancer, awareness, risk factors, surveys and questionnaires, Spain.

## Introduction

Oral cancer is considered a major public health problem, with variations in survival between countries and patient groups. Oral cancer represents the 11th most incident neoplasm (1), with over 202,000 with a male:female ratio 2:1 (2). In the particular case of Spain, oral cancer oral cancer ranks 16th among all neoplasms by incidence and 19th by mortality (3).

Variations in survival between and within countries are multifactorial and complex in nature, but a growing body of research suggests disease stage at the time of treatment could explicate some of them. Unfortunately, a large proportion of patients present with advanced disease (stages III and IV) mainly due to delay in self-referral (4).

Diagnostic delay in oral cancer has been found to be related to advanced stage at diagnosis and to influence patient survival (5), conditioned by the biological characteristics of the tumour.

Considering the limited improvements on survival rates to this neoplasm evidenced in the last decades despite the important technological advances in diagnosis and treatments, more attention is being paid to the events occurring since the first cancer-related symptom is experienced until healthcare is sought. This time interval represents a major component of waiting times since symptoms detection to definitive diagnosis of oral cancer (6), and it is reported to be associated to low awareness of cancer symptoms and risk factors (7).

Oral cancer is largely prevenTable (8) by avoiding known risk factors and adopting healthy lifestyles. In addition, the oral cavity is easily accessible for self-examination to detect suspicious lesions. Both approaches may have an impact on patient survival, but they clearly depend on the degree of patient awareness, which is reported to be very variable throughout Europe, ranging from the 96.6% of patients reporting they had heard of oral cancer in the UK in 2005 (9) to the 23.7% in the city of Porto (Portugal) (10). No information about oral cancer awareness in Spain could be retrieved beyond a pilot study undertaken by our group in a single city, which showed 22% of the participants had ever heard about oral cancer (11).

Several campaigns to increase oral cancer awareness have been undertaken in Spain throughout the years with apparently poor results. Although lack of information on cancer causes and knowledge on signs and symptoms has often been linked to a late diagnosis (10), raising awareness through this kind of campaigns seems to make little difference to the delay of patients seeking help (12).

Therefore, the aim of this investigation was to investigate public awareness of oral cancer in Galicia (NW Spain), as well as knowledge of risk factors, signs and symptoms.

## Material and Methods

This was a cross-sectional, community-based survey of randomly selected respondents from Galicia (North-western Spain) conducted from March 1, 2015 to 30 June 2016. The questionnaire was applied face-to-face by 14 specifically trained interviewers (postgraduate (n=7) and undergraduate dental students (n=2), 1 undergraduate medical student, 2 nurses, and 2 nurse assistants).

- Instrument development

We used a modification of the questionnaire originally developed by Rogers *et al* (12) in English language. The original instrument was translated into both Spanish and Galician and then back into English (double translation). Sociodemographic items in the instrument were adapted to the Galician environment, and an additional question on fruit intake was introduced in the questionnaire. The resulting questionnaire was piloted in a group of 5 clinicians and some items were reformulated, corrected, or deleted. This second draft was piloted in a group of 10 undergraduate dental students at the School of Medicine and Dentistry of the University of Santiago de Compostela and in a group of senior volunteers at a community centre of the Lugo city council.

- Participants and setting

Sample size was determined by quota sampling considering an accessible population of 5% and an expected percentage of response of 28% (12). The resulting sample size of 10,804 people permitted a power of 0.8% for estimating the proportion of oral cancer aware people, presuming a value of 25%.

Only people over 18 entered the study. The exclusion criteria were: (i) being mentally disabled and (ii) poor command of any of the official languages of the region (Galician or Spanish).

- Data collection

The study was undertaken in Galicia (North-western Spain), an autonomous region with 2,708,339 inhabitants unevenly distributed in 29,574.4 Km2, whose annual gross domestic product per capita is 21,358 € and their life expectancy at birth is 82.78 years. Data were obtained in all four capitals of the Galician provinces at four different areas in each city. These zones included administrative areas, and affluent and average-income commercial streets and shopping centres, in a sort of pathfinder survey method, according to the quota sampling procedure suggested by Rogers *et al* (12). The instrument was applied face-to-face in the community to randomly selected individuals who were approached by the interviewers in different week days and times at each location.

The interviewers participated in a 1 hour-long workshop which included discussion of the items in the instrument and their related ethical aspects, together with a role-playing session and a series of interviews to volunteer subjects (undergraduate dental students) under the supervision of a psychologist.

Data were coded and entered into a database. Each questionnaire was identified by a single number to permit an evaluation of the process of data coding and mechanization in a sample of randomly selected sets of data. Data were then transferred to statistical packages (R v3.3.2, MASS, and nnet) for analyses.

- Data analysis

Participants over 45, smokers and alcohol consumers, with a reported daily intake of less than 5 pieces of fruit per day were defined as “at risk” for the sake of data analysis.

A descriptive analysis was undertaken, and results presented as frequencies and percentages. Bivariate analysis was undertaken using the Chi Square/Fisher’s exact test. Results are expressed in terms of odds ratio with their 95% confidence intervals [OR (IC95%)]. Logistic regression analyses were also undertaken to disclose the variables influencing oral cancer awareness and to identify factors conditioning the recognition of main alarm signs. The level of significance chosen for all test was 5%.

- Ethical considerations

The study protocol was approved by the Santiago-Lugo Committee for Ethics in Research (number 2014/600). This investigation complied with the Spanish regulations and the Helsinki Declaration on ethical principles for medical research involving human subjects.

The results obtained from this research protocol are reported following the STROBE guidelines (Strengthening The Reporting of OBservational studies in Epidemiology) (13).

## Results

A total of 5,727 people accepted to participate in the survey (response rate: 53%). Participants were mostly in the 45-64 age group (30.2%; n=1,728), with a 47.7% of males (n=2,729).

- Oral cancer awareness

Participants were asked to mention all cancers they knew, and the first 10 responses were recorded. Breast (27.8%), lung (18.6%) and colorectal (12%) were the cancers most frequently mentioned as the first response. Oral cancer was mentioned by 3% of interviewees as their first response. It was recorded among the first three answers by 8.2% of the sample; 20.3% participants mentioned oral cancer amongst their responses in any position (Table 1). More than one third of participants (37.2%; n=415) had a relative or an acquaintance with oral cancer.

Active knowledge of oral cancer (unprompted mention) was shown by 1,024 individuals (17.95%). This percentage increased to about three quarters of the sample (73.1%; n=4,189) when specifically asked about this neoplasm (passive knowledge).

**Figure 1:**
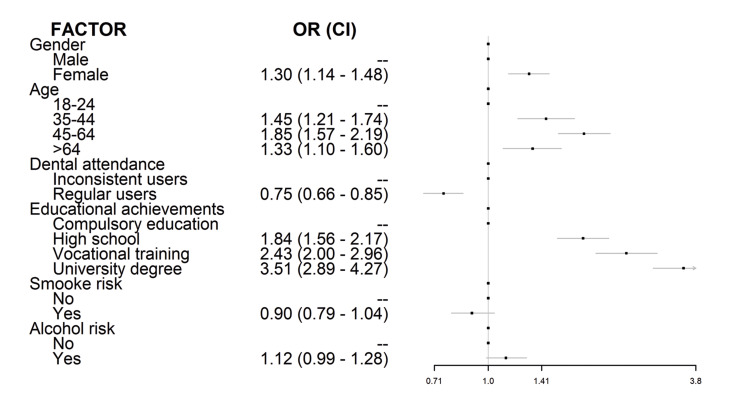
Factors influencing oral cancer awareness. Logistic regression analysis.

A logistic regression was performed (Fig. 1), and it was found that awareness had an OR=1.30 (1.14-1.48) in women regarding to men; we also found that all age ranges studied had a significant risk comparing to the reference category; and also that awareness increased with the educational level compared to compulsory education.

**Table 1:**
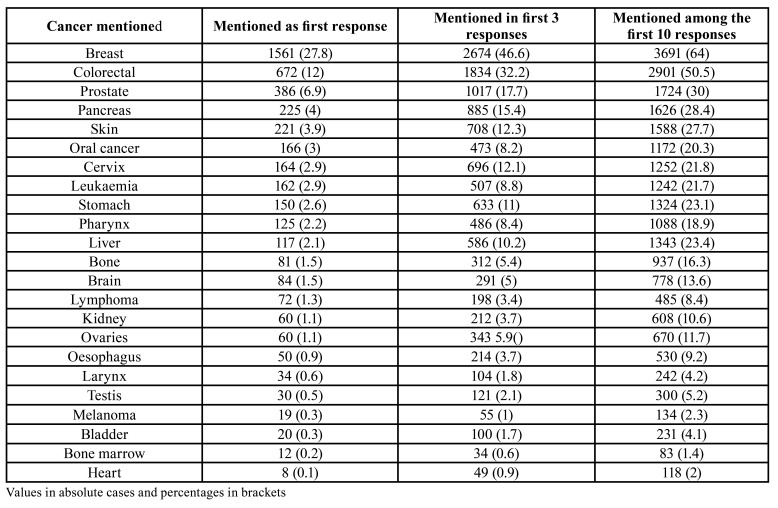
Cancers the sample had heard about mentioned in the first ten positions. Open, unprompted question.

- Preventive attitudes

Participants were asked about their daily intake of fruit and most of them reported to have 5 or more servings (pieces) of fruit per day (79.2%; n=4,538). When questioned about how frequently they attend their dentist, most interviewees (57.3%; n=3,281) reported to do it at least once a year. Regular dental visits were significantly associated with oral cancer awareness (77.9%; n=2,559 vs. 67.6%; n=1,576), with an adequate intake of fruits (23.4%; n=770 vs. 17.8%; n=416. *p*<0.001) and also with no smoking (59.9%; n=1,964 vs. 46.7%; n=1,092. *p*<0.001).

- Recognition of warning signs/symptoms

Responses on oral cancer symptoms (detailed in Table 2) ranked non-healing ulcerations as the most suggestive alarm sign, both prompted and unprompted, followed by mouth swelling as unprompted response, and sore tongue or mouth when prompted. Red or white patches gathered far lower percentages of participants connecting them with a possible oral neoplasm. Females recognized not-healing ulcerations as a potential symptom more frequently than their male counterparts (Table 3). Education also seems to have a part on this phenomenon: each step in the education ladder makes the participant 15% more likely of recognizing a red patch as an early cancer sign. In the case of white patches, each level beyond compulsory education increases the chances by one third the chances in the precedent level for acknowledging these lesions as potentially malignant. Volunteers with high school as their maximum scholar achievement elicited the highest chances for recognizing a non-healing ulceration as a suspicious sign, three-fold higher than those having completed vocational training courses.

- Knowledge about risk factors

Regarding active knowledge on oral cancer risk factors, the most frequently identified one was tobacco (55.3%; n=3,169), followed by alcohol (12.5%; n=708), poor oral hygiene (10.8%; n=618), diet (6.5%; n=377), and genetics (4.5%; n=248).

Current smokers resulted to be significantly more aware of the part of tobacco as a risk factor, a circumstance that does not occur with daily alcohol consumers who identified tobacco or alcohol as risk factors in lower percentages (Table 3). Progress in educational achievements ensures significantly more knowledge about oral cancer, as shown in Table 3.

A new variable was constructed in order to explore the knowledge individuals at high risk have about oral cancer. This subgroup of participants was defined by those over 45, current smokers and alcohol consumers, with a reported daily intake of less than 5 pieces of fruit per day. These people (7.5%; n=431) were mainly males, younger than 64 with compulsory education as their highest academic achievement (36.6%; n=158). Participants in the risk group were less aware or oral cancer, and this difference reached statistical signification in terms of active knowledge (Table 3). They also elicited differences in terms of recognition of potential cancer symptoms (Table 2).

**Table 2:**
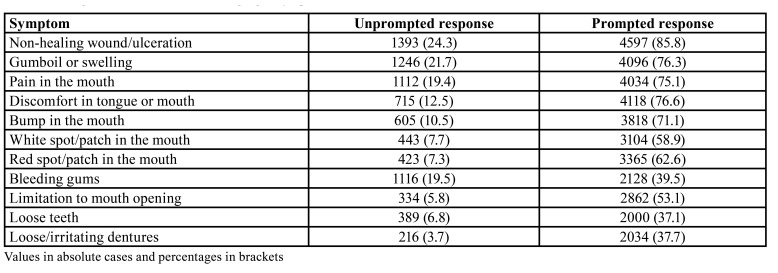
Recognition of oral cancer warning signs/symptoms.

**Table 3:**
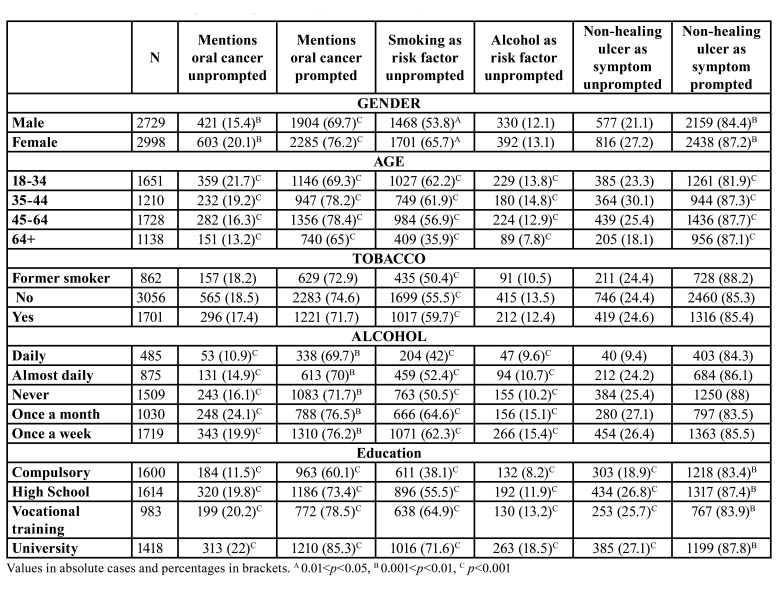
Characteristics of the sample and responses to key issues in the study.

**Figure 2:**
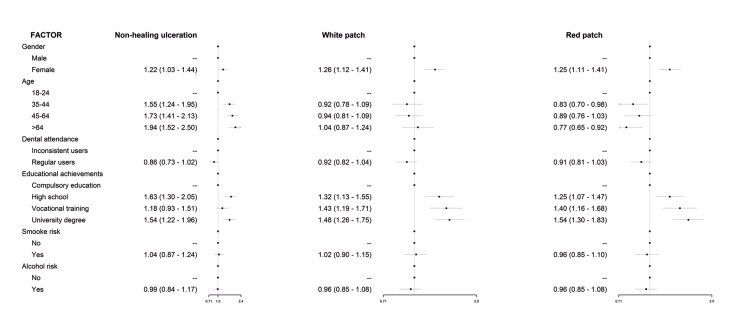
Factors influencing the recognition of main oral cancer warning signs/symptoms. Logistic regression analyses.

In order to gain insight into the features conditioning the recognition of the most relevant oral cancer signs/symptoms and risk factors, additional logistic regression analyses were undertaken (Fig. 2), resulting that females consistently recognize them better and that regular dental attenders perform worse than erratic users of dental services. The elder subgroup of participants is more likely to recognize tobacco and alcohol as risk factors or a red patch as an early sign of oral cancer than to identify white patches or non-healing ulcerations. Holding a university degree eases recognition of risk factors and early oral cancer signs with the exception of persistent ulcerations.

## Discussion

Our research approach permitted a reasonable balance of age and gender in the sample and a better feeling for people’s responses than is possible with a postal survey (12) or by telephone interview, with a clear advantage over this latter method given the growing number of homes using only mobile phones (14). Conversely, our study is limited by the fact of not having reached the intended sample size. In this circumstance, the precision of the study was recalculated for the sample size finally achieved [5,727] considering it an infinite population. It resulted a probability for a precision error of 1.16 in the estimation of a proportion by asymptotic 95% bilateral confidence interval, assuming an expected proportion of 28%. In addition, the recruitment method (at the busiest commercial and administrative areas in the four provinces of the region during several months at different times) and the participation of knowledgeable, specifically trained interviewers, may have contributed to counterweigh this drawback. However, the limitation of relying on self-reported data is inherent to this kind of studies (9) and may have influenced responses on habits and attitudes, as with knowledge items there is no objective criteria against which responses could be validated.

Another hypothetical bias may come from self-selection of participants, as those with lower health literacy may have declined the invitation to enter the study more frequently than other people. This phenomenon, if occurred, would only highlight the important deficit disclosed by our results.

The fact of having used the methodology suggested by Rogers *et al* (12) permitted interesting comparisons: 3% of our sample mentioned oral cancer in their first unprompted answer vs 1% in the Rogers’ group paper. The participants mentioning oral cancer in their first three or first ten responses (Table 1) double the percentages described in 2011 for the Mersey region (4% and 11% respectively) in England (12) but are far from the 56% reported for the whole Great Britain in 1999 through face-to-face interviews (15) or from the 95.6% identified by a postal survey undertaken all over Britain later in 2006 (9).

A similar study in the Portuguese city of Oporto in 2016 found that only 23.7% of the participants had heard of oral cancer (10), a finding that almost mimics our results (20.3%). Awareness can be related to prevalence, as persons should be more aware of the disorders more frequently found in their communities: oral cancer was ranked in 11th position by awareness by our sample while it is the 13th most incident cancer (including lip) in the region.

As occurred in previous studies (9,10,12-15), tobacco was the most frequently acknowledged risk factor (55.5%); the same as reported from Oporto (Portugal, 2015) (10), but far from the percentages reported from Northern Europe (84.7% in Great Britain [2006] (9); 76% in Schlesweiss-Holstein (Germany, 2012) (14); or 74% in the Mersey Region (UK, 2011)). Elder smokers recognized this risk less frequently: either they are less willing to accept their behaviour carries risk, or individuals recognizing the risk of smoking are more likely to stop (9). Our results indicate there is still a long way both in divulging the part of tobacco in oral cancer and in smoking cessation campaigns, particularly when current smokers are significantly more aware of the deleterious effect of tobacco smoking (10,12,16).

Alcohol consumption and its synergistic effect with tobacco smoking (17), seems to be less known to the public (10,14) as only 12.5% interviewees are aware this risk in contrast to the 19.4% reported for Great Britain (9), 21% for the Mersey region (12), to the 24.6% registered in Oporto (10); or the 50% in Schlesweiss-Holstein (14). This finding is particularly alarming, provided one quarter of participants reported to consume alcohol on a daily or almost daily basis. Alcohol consumers were more likely to identify alcohol intake as a risk factor than smoking. This may be a matter of concern, as could be the lower probability for regular dental attenders to recognize both risk factors.

Although few participants included diet among oral cancer risk factors, more than two thirds of the sample reported to consume five or more servings (pieces) of fruit per day. The Portuguese sample (11%) doubled our percentage of participants recognising the part of fruit intake in preventing oral cancer, but the number of people reporting to eat 5 pieces of fruit is three-fold larger in our sample. However, both samples are far from the 32% of Germans identifying a part for diet on risk for oral cancer (14).

Recognition of oral signs and symptoms is the start point in the pathways to treatment of oral symptomatic cancer. In this vein, the probability for recognizing early signs of oral cancer increases with age, with elder groups more likely to identify them. This apparently positive circumstance -oral cancer is largely a disease of elderly people (18)- does not apply to red patches, where participants over 64 are less likely to recognize this sign of alarm with higher risk for malignant transformation (19). The poor active knowledge on potential oral cancer symptoms -particularly white and red patches (9,10)- increased significantly when a response was prompted (12), but the low performance of individuals in the risk group may point at many precancerous lesions failing to be recognized along with opportunities to diagnose invasive carcinomas being missed (9).

The number of years of education completed has an effect on health-related outcomes (20). University graduates scored significantly higher percentages of correct answers (14). Although our survey did not analyse the socio-economic status of the participants -which has been linked to risk for oral cancer (21,22)- education is usually linked to employment and income (14) so our findings for the group with compulsory education as their highest educational achievement are particularly interesting.

Regular use of dental services also seems to have a negative influence on the recognition of the main oral cancer warning signs. Although it was not the aim of this study, and cross-sectional designs do not permit causal inferences, our results may well point at a low performance of dental clinics in educating patients for oral health (23) which may be worth of further investigation. Reasons for this low performance may include patient resistance, lack of time, lack of reimbursement mechanisms, and absence of readily accessible patient education materials (24).

The proportion of people aware of oral cancer significantly decreased in the elder group of participants (9,10,15). This finding, along with the known effects of age in health literacy, highlights the need for educational interventions specifically addressed to this population subgroup at increased risk. The use of awareness campaigns to promote early diagnosed of oral cancer can increase knowledge and the number of patients presenting at healthcare clinics in the short term (mainly those at lesser risk), but with limited evidence of long-term effectiveness (25). This seems to be valid for both individual and community-based interventions (26), with tailored printing information as the most effective medium for the former, and small groups and printed information for the latter. Mass-media campaigns have proved their usefulness in increasing cancer awareness (27), as well as the engagement of celebrities in delivering health-related messages (28).

In these circumstances, future oral cancer awareness programs should be tailored to the target audience and based on longer-term, multi-faceted approaches (25) that consider the social determinants of the disease and include adequate instruments for assessment. Systematised, opportunistic health education in clinical settings may also offer advantages over individual conventional approaches (29).

## Conclusions

It is concluded that general population has low awareness of oral cancer with poor knowledge of risk factors and main alarm signs. In addition, laypersons in the risk group scored lower values in the main variables analysed; even those highly educated showed insufficient awareness and knowledge of oral cancer. Thus, there is a clear need for educational interventions tailored to the target audience and aimed at increasing knowledge and awareness of oral cancer to promote primary prevention of oral cancer and minimising the time interval of patients with symptomatic oral cancer in their path to treatment.
